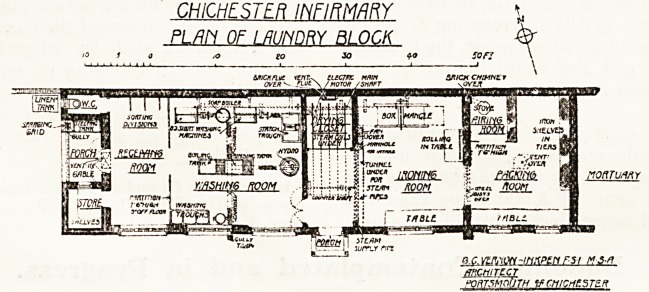# Chichester General Infirmary: The New Laundry

**Published:** 1912-11-23

**Authors:** Tom B. Harrison

**Affiliations:** Secretary.


### Chichester General Infirmary: The New Laundry.

By TOM B. HARRISON, F.I.S.A., Secretary.

FOR some years past the laundry of the above institution has been in a most antiquated state, almost everything in the way of plant being of a most obsolete and out-of-date pattern, and taking into consideration the type of machinery in use, the good work done up to the time of the new laundry being built was really wonderful. The board of management realised that it was absolutely necessary that something should be done in the matter of reconstructing the old laundry and bringing same up to date. It was thought that the work might be included in the present reconstruction scheme of the main building, but happily, owing to the generosity of a kind and generous donor, who gave a sum of £1,030 to provide and equip a new laundry, the board were relieved of the anxiety of providing the same out of the main building fund, and which probably meant a consequent loss of some useful addition to the main building.

The architect appointed for the work of carrying out the scheme was asked to submit plans for a new laundry, providing for the washing of, say, 2,500 pieces per week, which should not entail a greater expenditure than £1,030, such work to include all architect's fees, fees for quantities and supervision of work, all builders' inclusive costs, cost of machinery and fixing same, drainage and all subsurface work, and cost of any other work in connection with the undertaking, and an allowance of 2½ per cent. on the total outlay for contingencies, a most important item.

The Infirmary contains 60 beds.

### Structural.

The old laundry consisted of washing, ironing, and airing rooms, the two former being one storey high, and the airing room, a two-storey building which was formerly an old-fashioned brew-house, with the floor above removed. Little or no accommodation was provided for sorting and dealing with articles to be washed or for packing and sorting the clean linen. The floors were of stone, which had worn unevenly, and the different rooms were not on the same level, thus considerably restricting the easy transfer of heavy baskets of linen from one department to another. The old laundry has now been extended about 28 feet at its western end, the floors have been levelled throughout, and the roofs reconstructed and reduced to one level from end to end. In designing the arrangement of the new laundry care has been taken to maintain a proper cycle of operations. The work is received at the western end of the laundry, the foul linen requiring special attention being treated in a steeping tank with sparging grid and gully (which also serves for washing-down purposes) situated in the open porch. A receiving room, with movable wire sorting divisions, is next provided, separated from the main washing room by a partition; the walls of both the receiving and washing rooms are faced with white glazed bricks to a height of 5 feet all round. The whole of the floors of the receiving and washing rooms are covered with asphalte, with rounded angles and skirting of the same material; suitable channels covered with iron gratings are provided in the floor for taking the drainage from machines and surface.

The ironing room was designed sufficiently large to accommodate a Decoudin ironing machine, but for financial reasons and the comparatively small output of the laundry a power-driven box mangle is temporarily installed in lieu thereof. The floors of the ironing, airing, and packing rooms are of stone taken from the old building refaced. The walls of these rooms are treated with a painted dado, and colour-washed from dado to ceiling, which consists of varnished match-boarding throughout. The airing and packing rooms are arranged close together, separated by a plaster and metal lathing partition, and having open wire shelves in tiers and packing tables. Ventilation is maintained by an electric fan situated in the gable, also an extract ventilator in the roof, and suitably arranged inlets.

### Machinery.

The old machinery and appliances comprised a rotary hand-washing machine, blueing and rinsing troughs, three fairly up-to-date Doulton's washing troughs, and a hand-operated box mangle; drying was effected by means of an obsolete pattern steam-heated drying chamber with run-in horses, and the average output of the laundry was 1,800 pieces per week. In the new laundry the machinery in the washing room comprises two 80 shirt-washing machines, soap boiler, starching trough, a large boiling and rinsing tank, also a hydro, calorifier, and the three present washing troughs refixed. A new steam coil heated five-horse drying closet is provided, the steam being generated in the boiler house situated in the main building. Electrically driven fans circulate the air in the drying chamber. The whole of the machines are driven by a 5 h.p. electric motor running at 2,500 revolutions per minute, the power being communicated to the various machines by means of counter shafting and belt drive. Water for washing purposes is obtained from a large rain-water storage tank, a relic of the old brew-house, which exists beneath the old buildings, and which has come in extremely useful, probably saving the cost of purchasing a water-softening appliance.

Messrs. Bradford and Co., of Manchester and London, installed the machinery, and Messrs. Vick and Son, of Chichester, were the builders, under the direction of the architect, Mr. G. C. Vernon Inkpen, of Portsmouth and Chichester.

ADMINISTRATIVE.

The laundry is staffed as follows: One head laundress, one assistant laundress (who both live in), and a woman scrubber, who is employed daily at 2s. 6d. per day, without emoluments. This staff has proved to be quite adequate, and is a saving compared with the old laundry, when an extra woman was brought in twice a week. The porters carry the linen backwards and forwards from the laundry. The weekly allowance of cleaning materials, etc., is as follows: Soap 12 lb., soda 26 lb., starch 5 lb., borax $1\frac{1}{2}$ lb., blue $\frac{1}{2}$ lb.; about 5 cwt. coke and $1\frac{1}{4}$ cwt. coal is used weekly for ironing stove, etc. The current used by the electric motor is obtained at a special power rate of $1\frac{1}{4}$d. per unit, and amounts to 2s. 6d. per week on the average.

### OUR EXPERT'S COMMENTS ON THE CHICHESTER INFIRMARY LAUNDRY.

Granted that a laundry is a necessary adjunct to a hospital of less than 100 beds, then the arrangements of the Chichester laundry appear to be satisfactory. It is to be supposed that the site governs the general shape of this laundry, otherwise a rather squarer design would have been preferable. For the size of the hospital the area of the laundry is ample. Doubtless the architects have had an eye to increase. The drying closet is well placed, but compared with the rest of the laundry it seems to be on the small side. We notice at the entrance a sparging grid and a steeping tank. We strongly hold that fouled linen should be roughly cleaned in the wards by the nurses. It is an objectionable practice to allow articles to leave the ward in a very foul condition. It may be, of course, that in the Chichester Infirmary the wards have not the necessary appliances. If that is so then they have no option in the matter. We presume that the laundry is to be used for patients and staff. If so there is no provision for any separation in the receiving and washing room. It may be that the committee consider this a counsel of excellence in a small hospital, and no doubt where space is ample and the work not overwhelming there is not the same necessity for separation as in a laundry which is worked to its fullest capacity; still as a general principle there is every reason why the staff and the patients' washing should be kept separate, and if there is not the same necessity for this in the laundry of a small hospital as in the laundry of a larger, then this rather goes to show that it is a question whether a hospital of less than 100 beds should have its own laundry.

We notice that the building has windows on two of its four sides only. We hope that the architect has made ample provision for getting rid of the hot air and steam by top ventilation. A stuffy laundry is an abomination and tells on the workers.

## Figures and Tables

**Figure f1:**